# Clinical characteristics of adrenal crisis in adult population with and without predisposing chronic adrenal insufficiency: a retrospective cohort study

**DOI:** 10.1186/s12902-017-0208-0

**Published:** 2017-09-11

**Authors:** Masahiro Iwasaku, Maki Shinzawa, Shiro Tanaka, Kimihiko Kimachi, Koji Kawakami

**Affiliations:** 1 Department of Pharmacoepidemiology, Graduate School of Medicine and Public Health, Kyoto University, Yoshida-Konoe-cho, Sakyo-ku, Kyoto, 606-8501 Japan; 2 Department of Healthcare Epidemiology, Graduate School of Medicine and Public Health, Kyoto University, Yoshida-Konoe-cho, Sakyo-ku, Kyoto, 606-8501 Japan

**Keywords:** Adrenal crisis, Adrenal insufficiency, Relative adrenal insufficiency, Glucocorticoid, Cohort study

## Abstract

**Background:**

Adrenal crisis (AC) occurs in various clinical conditions but previous epidemiological studies in AC are limited to chronic adrenal insufficiency (AI) and sepsis. The aim of this study was to investigate characteristics of AC patients, including predisposing diseases and to describe candidate risk factors for AC such as comorbidities and glucocorticoid (GC) therapy.

**Methods:**

We conducted a retrospective cohort study using a claims database on 7.4 million patients from 145 acute care hospitals between January 1, 2003 and April 30, 2014. We identified AC patients who met the following criteria: 1) disease name with ICD-10 corresponded with AI; 2) therapeutic GC administration (hydrocortisone equivalent dose ≥100 mg/day); 3) admission; and 4) age ≥18 years.

**Results:**

We identified 504 patients with AC (median age, 71 years; interquartile range, 59 to 80; 50.6% male). As predisposing conditions, primary AI and central AI accounted for 23 (4.6%) and 136 patients (27.0%), respectively. In the remaining AC patients (68.5%), comorbidities such as cancer, autoimmune diseases, and renal failure were frequent. The most frequent indication for hospitalization was AC (16.3%), followed by pituitary disease (14.7%), cancer (14.7%), AI-related clinical symptoms (11.5%), and infection (11.1%). Admission under oral GC treatment was reported in 104 patients (20.6%). Twenty-six patients were admitted within 14 days after GC cessation (5.2%).

**Conclusions:**

These findings present an overview of patients with AC in general practice settings, clarifying that predisposing factors for AC were complicated and that patients other than those with chronic AI were older and had more comorbid conditions than those with primary and central AI.

**Electronic supplementary material:**

The online version of this article (10.1186/s12902-017-0208-0) contains supplementary material, which is available to authorized users.

## Background

Adrenal crisis (AC) has a rapid course and can be life threatening, requiring prompt treatment such as administration of glucocorticoids (GC) [1]. Endocrine laboratory values have not been established for the diagnosis of AC [2]; the diagnosis of AC is based on clinical symptoms and the patient’s general condition [3–5].

Major risk factors for AC include chronic adrenal insufficiency (AI), infection, and administration of exogenous GC [1, 3–5]. The association of chronic AI with AC has been well studied although this condition is relatively rare [1, 3, 4]. In contrast to AC, chronic AI can be diagnosed by hormone testing [1]. Severe infection can be accompanied by relative AI, which is an inadequate cortisol response relative to the severity of the patient’s illness and is similar to AC [6]. The Surviving Sepsis Campaign Guidelines suggested optional administration of intravenous hydrocortisone for relative AI without precise indications that would require administration of hydrocortisone [2]. GC is one of the most prescribed drugs [7] and has been reported as the most common cause of AI [8]. Comorbidities, surgery, and aging have also been related to the incidence and morbidity of AC [4, 5, 9, 10].

Previous studies of AC, including relative AI, focus on specific populations such as those with chronic AI or sepsis [2–5, 9, 11]. Therefore, characteristics of patients differ among studies and application of the knowledge acquired is complicated. Administrative databases have been used recently for clinical studies of AC [9, 10]. To overcome limitations related to the specificity of study populations and treatment settings, in the present study we also used a large medical database from acute care hospitals that included all admissions to these hospitals. The objective of the study was to investigate and compare characteristics of patients with AC in accordance with predisposing diseases and to describe candidate risk factors such as comorbidities and GC therapy.

## Methods

### Data source

This is a retrospective cohort study using a multihospital claims database provided by Medical Data Vision Co., Ltd. (Tokyo, Japan). As of April 2014, the database consisted of information on 7.39 million patients from 145 diagnosis procedure combination (DPC) hospitals across Japan and covered about 9% of all acute care hospitals located in multiple districts. DPC hospitals are acute phase hospitals administered under the Diagnosis Procedure Combination/Per-Diem Payment System. The database has two parts, i.e. administrative data on outpatients and inpatients and in-hospital data on DPC [12]. Items in the database included patient characteristics (age, sex), medical conditions (disease indicating hospital admission, comorbidities on admission, primary disease, secondary disease, and post-admission complications) which were classified according to the International Statistical Classification of Diseases and Related Health Problems 10th Revision (ICD-10) and medical care provided (prescriptions, laboratory tests, interventional procedures such as surgery, date of admission) [13, 14]. For this database, attending physicians were in charge of recording the diagnoses through referring to medical charts. Age and gender distributions of the source population were similar to those of the national census in Japan and several epidemiologic studies using the database had been published [14, 15]. We analyzed de-identified data entered between January 1, 2003 and April 30, 2014 for this study. This study was approved by the Kyoto University Graduate School and Faculty of Medicine, Ethics Committee (E2051). This study was exempt from obtaining individual informed consent based on the Ethical Guidelines for Medical and Health Research Involving Human Subjects by the Ministry of Health, Labour, and Welfare and Ministry of Education, Culture, Sports, Science and Technology.

### Study cohort and disease definition

AC is a potentially life-threatening condition and needs immediate emergency treatment. In general, it is more severe than AI, but there are no clear distinctions between them. To identify appropriate cases of AC, we used a two-step process. First, we identified candidate patients using ICD-10 in combination with a disease name that corresponded to AI (Additional file 1: Table S1). Both were recorded under administrative data consistent with AI, and those patients were enrolled in the study. We assumed that the date of recording AI was the date of diagnosis; therefore, we used that date as the index date. Second, we confirmed AC in the study cohort according to fulfillment all of the following criteria: 1) therapeutic GC administration (hydrocortisone equivalent dose ≥100 mg/day) within 3 days before or after the index date (Additional file 2: Figure S1); 2) admission within 3 days before or after the index date (Additional file 2: Figure S2); and 3) age 18 years or older at the index date.

We did not provide an exclusion criterion for treatment with high-dose GC (hydrocortisone equivalent dose >200 mg/day) because we intended to investigate practical clinical management of patients with AC. Besides, we considered the possibility that hospitals stocked a variety of dosages. Treatment with excessive GC administration may be inappropriate for AC, we assessed separately for patients receiving excessive GC administration (hydrocortisone equivalent dose >1000 mg/day) (Additional file 1: Table S2).

### Predisposing conditions

Hypothalamic-pituitary-adrenal (HPA) axis dysfunction may be a risk factor for AC. We defined and classified predisposing conditions according to the pathologic mechanism of HPA axis function. The predisposing conditions were classified as either primary or central AI or “Others”.

Primary AI results from a disease intrinsic to the adrenal cortex. There are two major mechanisms for the development of primary AI: 1) adrenal hypofunction such as in Addison’s disease and 2) medical interventions for adrenal disease, such as surgical resection of the bilateral adrenal cortex, leading to adrenal hypofunction. Additional file 1: Table S3 and Table S4 show diseases and interventions that are risk factors for primary and central AI based on previous reports [1, 16]. For this study, we determined that patients with primary AI had to meet either of the following criteria: 1) the presence of a disease considered to be the cause of primary AI and 2) having had an adrenal disease for which an intervention involving the adrenal cortex was performed twice or more since the adrenal organ exists bilaterally.

Central AI results from impairment of the hypothalamic-pituitary axis. We identified patients with central AI by either the presence of central AI itself or having undergone an intervention involving the hypothalamic-pituitary axis at least once.

There could be a prolonged lag between the hospital visit and diagnosis of predisposing diseases [17, 18]. We collected information on predisposing conditions from administrative data recorded before the admission and during the hospitalization.

The category of “Others” included various predisposing diseases, examples of which are undiagnosed chronic AI, relative AI, and drug-induced AI. But, provision of criteria is difficult for these specific predisposing conditions. To explore these latent conditions in “Others”, we compared clinical characteristics on admission with or without the following: 1) prior admission (within 1 year before AC); 2) GC medication (including drugs interacting with GC), and 3) GC cessation (within 30 days before AC).

### Disease indicating hospital admission and comorbidities on admission

Indications for hospital admission are based on a clinical diagnosis leading a physician to decide on hospitalization. Such indications would include clinical symptoms related to AI (Additional file 1: Table S5). We defined clinical symptoms related to AI based on published data [1]. From the database, we identified the following comorbidities on admission by using DPC data: cardiovascular disease, infection, sepsis, cancer, diabetes mellitus, hypothyroidism, any autoimmune disease, peptic ulcer, chronic obstructive pulmonary disease or asthma, renal failure, osteoporosis, dementia, and liver disease (Additional file 1: Table S6). In selecting these comorbidities, we referred to previous studies to confirm a possible relationship with AC [19–22].

### Determination of drug and treatment regimens

To identify medications, we complied with the European Pharmaceutical Marketing Research Association (EphMRA) Anatomical Guidelines. We determined GC drugs corresponding with the H2 category, which indicates systemic corticosteroids in EphMRA. To accurately calculate the dose administered, we excluded uncountable administrations such as by a nebulizer since with this method of administration it is not possible to precisely estimate absorption. Our study did not count fludrocortisone as a GC because fludrocortisone had not been used for an anti-inflammatory effect (Additional file 1: Table S7). Additionally, we did not use fludrocortisone as a proxy of GC replacement therapy for primary AI. Japanese practice guidelines did not recommend fludrocortisone as a routine replacement therapy for primary AI because the Japanese adult population ingests a relatively large amount of salt [23].

We defined the occurrence of AC under treatment with oral GC if the index date occurred while the patient was being prescribed oral GC. Regarding inhaled GC and intra-nasal GC, we determined that AC had occurred while under treatment if the last prescription was within 90 days before the index date.

Concomitant administration of interacting drugs could modify GC effects and metabolism. As previous studies reported the interaction of various drugs with GC [1, 17, 24], we checked such medications using the combination of the EphMRA code and the actual drug name (Additional file 1: Table S8). Determination of the occurrence of AC under treatment with interacting drugs was defined by the same protocol as used for oral GC treatment.

### Sensitivity analysis

To identify the study cohort more rigorously, we made an alternative (“narrowly defined”) case definition based on laboratory tests and clinical decisions. For selecting a narrowly-defined case, we required two additional restrictions: 1) having in-hospital hormone testing (cortisol, adrenocorticotrophic hormone, endocrine stimulation test, or adrenal cortex stimulation test) and 2) AI was recorded in DPC data along with any information that included primary, secondary, or a disease indicating admission, comorbidity on admission, or post-admission complication. Hormone testing is not necessary for diagnosis of AC, but Japanese practice guidelines recommended that any condition suspicious for AC should be treated immediately after taking the blood sample (cortisol, adrenocorticotrophic hormone) [23].

### Statistical analyses

Characteristics of the study population were summarized using proportions for categorical variables and the median and interquartile range for continuous variables.

## Results

### Patients

**Fig. 1:**
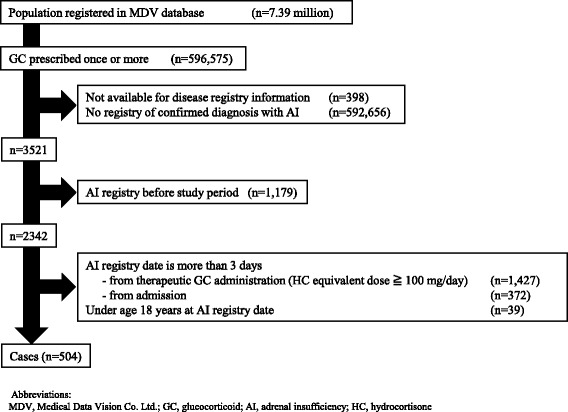
Flow diagram of identification of patients with adrenal crisis

We obtained a claims database that included 7.39 million patients and identified 504 AC patients who met both inclusion and exclusion criteria (Fig. 1). The characteristics of the study population are shown in Table 1. Median age was 71 years (interquartile ranges [IQR], 59 to 80), and half of the participants were male (50.6%). The most common reporting department was internal medicine (50.6%) followed by neurosurgery (18.7%) and urology (3.6%). AC tended to occur in colder seasons of the year: Jan – March, 29.6%; April – June, 22.2%; July – Sept, 22.2%; Oct – Dec, 26.0%.

**Table 1:** Demographic and clinical characteristics of patients with adrenal crisis at admission

		ALL
		(*n* = 504)
Age(years)	Median(IQR)	71 (59–80)
	<40 yr	47 (9.3)
	≧40 yr. <60 yr	88 (17.5)
	≧60 yr. <80 yr	236 (46.8)
	≧80 yr	133 (26.4)
Male		255 (50.6)
Disease	Adrenocortical insufficiency	264 (52.4)
	Adrenal crisis	194 (38.5)
	Secondary adrenocortical insufficiency	28 (5.6)
	Post-procedural adrenocortical hypofunction	10 (2.0)
	Steroid withdrawal syndrome	5 (1.0)
	Iatrogenic adrenocortical insufficiency	3 (0.6)
Department	Internal medicine	255 (50.6)
	Neurosurgery	94 (18.7)
	Urology	18 (3.6)
	Others	137 (27.2)
Season	Jan - March	149 (29.6)
	April–June	112 (22.2)
	July - Sept	112 (22.2)
	Oct - Dec	131 (26.0)

*Abbreviation*: *IQR* Interquartile

As for predisposing conditions, primary AI was diagnosed in 23 (4.6%) such conditions and central AI was diagnosed in 136 (27.0%). The “Others” category included those with various pathophysiologic backgrounds. Patients in the “Others” category were older and had more comorbidities such as cancer, autoimmune diseases, or renal failure.

### Disease indicating hospital admission and comorbidities on admission

As shown in Table 2, for patients with primary AI, the most frequent indication for hospitalization was AC (26.1%). Half of patients with central AI were hospitalized for pituitary disease (50.7%). Among other responsible diseases for admission, in the “Others” category cancer was the most prevalent (20.0%). Infectious diseases, including infection and sepsis, accounted for 14.5% of all admissions. In a substantial proportion of cases, AI-related clinical symptoms were indications for hospitalization.

**Table 2:** Clinical characteristics of patients with adrenal crisis at admission separated by different pathophysiological mechanisms

		Primary AI	Central AI	Others	ALL
		(*N* = 23)	(*N* = 136)	(*N* = 345)	(*n* = 504)
Age (years)	Median (IQR)	59 (36–73)	65 (49–74)	73 (62–82)	71 (59–80)
Male		9 (39.1)	73 (53.7)	173 (50.1)	255 (50.6)
Indication for hospital admission^a^	Adrenal insufficiency^b^	6 (26.1)	26 (19.1)	50 (14.5)	82 (16.3)
	Pituitary disease	3 (13.0)	69 (50.7)	2 (0.6)	74 (14.7)
	Cancer	3 (13.0)	2 (1.5)	69 (20.0)	74 (14.7)
	Infection	2 (8.7)	10 (7.4)	44 (12.8)	56 (11.1)
	Cardiovascular disease	0 (0.0)	2 (1.5)	19 (5.5)	21 (4.2)
	Sepsis	1 (4.3)	3 (2.2)	13 (3.8)	17 (3.4)
	Adrenal tumor	0 (0.0)	0 (0.0)	10 (2.9)	10 (2.0)
	AI-related clinical symptom^c^	2 (8.7)	12 (8.8)	44 (12.8)	58 (11.5)
Comorbidity^d^	Cardiovascular disease	7 (30.4)	40 (29.4)	109 (31.6)	156 (31.0)
	Infection	5 (21.7)	26 (19.1)	65 (18.8)	96 (19.0)
	Diabetes	1 (4.3)	22 (16.2)	57 (16.5)	80 (15.9)
	Cancer	1 (4.3)	0 (0.0)	66 (19.1)	67 (13.3)
	Hypothyroidism	2 (8.7)	27 (19.9)	24 (7.0)	53 (10.5)
	Autoimmune disease	0 (0.0)	1 (0.7)	32 (9.3)	33 (6.5)
	Peptic ulcer	2 (8.7)	9 (6.6)	21 (6.1)	32 (6.3)
	COPD or asthma	1 (4.3)	4 (2.9)	21 (6.1)	26 (5.2)
	Renal failure	0 (0.0)	5 (3.7)	21 (6.1)	26 (5.2)
Therapeutic GC regimen	HC	18 (78.3)	107 (78.7)	203 (58.8)	328 (65.3)
	mPSL	4 (17.4)	14 (10.3)	56 (16.2)	74 (14.7)
	DEX	1 (4.3)	6 (4.4)	37 (10.7)	44 (8.7)
	Others	0 (0.0)	9 (6.6)	49 (14.2)	58 (11.3)

*Abbreviations*: *AI* adrenal insufficiency, *IQR* interquatile, *COPD* chronic obstructive pulmonary disease, *GC* glucocorticoid, *HC* hydrocortisone, *mPSL* methylprednisolone, *DEX* dexamethasone

^a^Identified as disease or symptom requiring admission decision, registered according to the Japanese diagnostic procedure combination (DPC) system

^b^Considering the following admission and therapeutic GC administration, this group is consistent with hospitalization due to adrenal crisis

^c^Consisted of unspecified coma, hyponatremia, unspecified hypotension, volume depletion, shock, anorexia, nausea, vomiting, unspecified fever and hypoglycemia

^d^Identification of comorbidity is based on comorbidity lists at admission, registered according to the Japanese diagnostic procedure combination (DPC) system

Regarding comorbidities, cardiovascular disease was the most frequent in this study population (31.0%), followed by infection (19.0%), diabetes (15.9%), cancer (13.3%), and hypothyroidism (10.5%). Prevalence of several comorbidities was also associated with predisposing diseases. Hypothyroidism was often reported in central AI (19.9%). Cancer, autoimmune diseases, and renal failure were more frequent in patients with “Others” (19.1%, 9.3%, and 6.1%, respectively). Liver disease, osteoporosis, and dementia were little observed in this study population (2.8%, 1.6%, and 1.2%, respectively).

A total of 110 patients (21.8%) received an in-hospital operation under general anesthesia (55 with central AI, 5 with primary AI, and 50 with “Others”). Most of the operations performed within 3 days after admission (101 patients, 91.8%).

### Therapeutic glucocorticoid administration

**Fig. 2:**
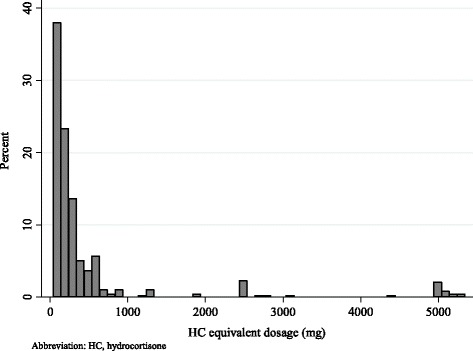
Peak daily dosage of intravenous glucocorticoids

Hydrocortisone was most often selected as a regimen of therapeutic GC (65.3%), especially for primary AI (78.3%) and central AI (78.7%). Peak daily dosage of intravenous GC is shown in Fig. 2. Median dosage was equivalent to 200 mg (IQR, 100 to 352) of hydrocortisone per day.

### Hormone testing

The serum cortisol level was measured in hospital in 258 patients (51.2%) (Additional file 2: Figure S3). Few patients had undergone the adrenal cortex stimulation test (21patients, 4.2%). Patients with central AI tended to have more frequent hormone testing (cortisol 101 patients, 74.3%; adrenal cortex stimulation test 11 patients, 8.1%).

### Prior treatment before onset of adrenal crisis

Two fifths of patients had been referred to acute care hospitals and required hospitalization for AC (219 patients, 43.5%). Two thirds of AC patients had visited the hospital within 90 days before the onset of AC (337 patients, 66.9%) (Table 3). When the time frame was limited to within 14 days, the number of patients was 241 (47.8%). Admission under oral GC treatment was reported in 104 patients (20.6%). Median dosage of oral GC treatment on admission was equivalent to 20 mg of hydrocortisone per day. No patient was hospitalized under treatment with intranasal GC. Fifteen patients (3.0%) were treated with inhaled GC and were mostly patients specified as “Others” (14 patients). Treatment to modify the effect of GC was reported in a small number of patients (13 patients, 2.6%).

**Table 3:** Patients who visited hospital before admission or were admitted while under GC-related medication

		Primary AI	Central AI	Others	ALL
		(*N* = 23)	(*N* = 136)	(*N* = 345)	(*n* = 504)
Visited before admission	Yes	18 (78.3)	108 (79.4)	258 (74.8)	384 (76.2)
	Yes, within 90 days before	15 (65.2)	98 (72.1)	224 (64.9)	337 (66.9)
	Yes, within 14 days before	11 (47.8)	69 (50.7)	161 (46.7)	241 (47.8)
Under GC-related medication	Oral GC	5 (21.7)	31 (22.8)	68 (19.7)	104 (20.6)
	HC equivalent daily dosage (mg)	15 (10–20)	20 (10–22.5)	20 (20–40)	20 (10–40)
	Median [IQR]				
	Intranasal GC	0 (0.0)	0 (0.0)	0 (0.0)	0 (0.0)
	Inhaled GC	0 (0.0)	1 (0.7)	14 (4.1)	15 (3.0)
	Drug that interacts with GC	2 (8.7)	3 (2.2)	8 (2.3)	13 (2.6)

*Abbreviations*: *IQR* Interquartile range, *HC* hydrocortisone, *GC* glucocorticoid

Cessation of exogenous GC administration could be a risk factor for AC. We counted the time lags between admission and the previous GC cessation. Admission within 14 days after GC cessation was observed in 26 patients (5.2%), and when the time from cessation to admission was extended to within 30 days 36 patients were identified (7.1%).

### Characteristics of patients in the “others” group

There was a higher proportion of patients in the “Others” group than in the Primary AI and Central AI groups with admissions within 1 year before AC (Primary AI: 17.4%, 4/23; Central AI: 21.3%, 29/136; Others: 44.1%, 152/345). We compared characteristics of patients in “Others” according to the presence of the following groups: 1) prior admission (Additional file 1: Table S9); 2) GC medication (Additional file 1: Table S10), and 3) GC cessation (Additional file 1: Table S11). Patient characteristics were similar among those in the three groups: cancer was a major comorbidity and hormone testing before AC had been performed widely. Prior admission within 1 year before AC was prevalent in patients in either GC medication group or GC cessation group (65.0% or 90.3%, respectively). One key difference between these two groups was that the patients in GC cessation group did not include any patient with an autoimmune disease as a comorbidity.

### Sensitivity analysis

Table 4 shows results of the sensitivity analysis using different disease definitions, namely, whether the 504 patients were further classified as having AC according to the more strict criteria termed as “narrowly-defined cases.” The narrowly defined cases included more central AI patients than the non-narrowly defined cases (37.3% vs. 20.1%).

**Table 4:** Clinical characteristics at admission of narrowly defined cases of AC and non-narrowly defined cases of AC

		Narrowly defined case	Non-narrowly defined case
		(*n* = 201)	(*n* = 303)
Age(years)	Median [IQR]	72 (60–81)	70 (57–79.5)
	<40 yr	18 (9.0)	29 (9.6)
	≧40 yr. <60 yr	31 (15.4)	57 (18.8)
	≧60 yr. <80 yr	95 (47.3)	141 (46.5)
	≧80 yr	57 (28.4)	76 (25.1)
Male		102 (50.7)	153 (50.5)
Predisposing disease	Primary AI	7 (3.5)	16 (5.3)
	Central AI	75 (37.3)	61 (20.1)
	Others	119 (59.2)	226 (74.6)
Indication for hospital admission^a^	Adrenal insufficiency^b^	52 (25.9)	30 (9.9)
	Pituitary disease	41 (20.4)	33 (10.9)
	Cancer	7 (3.5)	67 (22.1)
	Infection	15 (7.5)	41 (13.5)
	Cardiovascular disease	7 (3.5)	14 (4.6)
	Sepsis	6 (3.0)	11 (3.6)
	Adrenal tumor	4 (2.0)	6 (2.0)
	AI-related clinical symptom^c^	40 (19.9)	18 (5.9)
Therapeutic GC regimen	HC	149 (74.1)	179 (59.1)
	mPSL	24 (11.9)	50 (16.5)
	DEX	15 (7.5)	29 (9.6)
	Others	13 (6.5)	45 (14.9)
Hormone testing	ACTH	178 (88.6)	56 (18.5)
	Cortisol	194 (96.5)	64 (21.1)
	Endocrine stimulation test^d^	27 (13.4)	13 (4.3)
	Adrenal cortex stimulation test	15 (7.5)	6 (2.0)
Visited before admission	Yes, within 90 days before	124 (61.7)	213 (70.3)
	Yes, within 14 days before	90 (44.8)	151 (49.8)
Under GC-related medication	Oral GC	33 (16.4)	71 (23.4)
	HC equivalent daily dosage (mg)	20 (12–30)	20 (20–40)
	Median [IQR]		
	Intranasal GC	0 (0.0)	0 (0.0)
	Inhaled GC	8 (4.0)	7 (2.3)
	Drug interacting with GC	4 (2.0)	9 (3.0)

*Abbreviations*: *AI* adrenal insufficiency, *GC* glucocorticoid, *HC* hydrocortisone, *mPSL* methylprednisolone, *DEX* dexamethasone, *ACTH* adrenocorticotropic hormone, *IQR* Interquartile.

^a^Identified as disease or symptom requiring decision to admit registered in the DPC system

^b^Considering the following admission and therapeutic GC administration, this group is consistent with hospitalization due to adrenal crisis

^c^Consisted of unspecified coma, hyponatremia, unspecified hypotension, volume depletion, shock, anorexia, nausea, vomiting, unspecified fever and hypoglycemia

^d^Endocrine stimulation test consists of hormone dynamic testing of the following: anterior pituitary (growth hormone, gonadotropin, thyroid stimulating hormone, prolactin, adrenocorticotropic hormone), posterior pituitary (antidiuretic hormone), thyroid, parathyroid, and gonad (testosterone, estradiol). Endocrine tests of adrenocorticotropic hormone included insulin tolerance test, metyrapone test, dexamethasone suppression test, and corticotropin-releasing hormone stimulation test. In this study, we counted results of the adrenal cortex stimulation test apart from those for endocrine stimulation tests. The adrenal stimulation tests evaluates adrenal cortex function, which is related to glucocorticoid or mineralocorticoid, for example, the adrenocorticotropic hormone stimulation test

Disease indicating admission differed between the two groups. Endocrine disease and AI-related clinical symptoms were more prevalent in the narrowly defined cases than in the non-narrowly defined group as follows: adrenal crisis, 25.9% vs. 9.9%; pituitary disease, 20.4% vs. 10.9%; and AI-related clinical symptoms, 19.9% vs. 5.9%. Cancer and infection were less prevalent in the narrowly defined cases as follows: cancer, 3.5% vs. 22.1%; infection, 7.5% vs. 13.5%. Hydrocortisone was selected as the therapeutic regimen more frequently in the narrowly-defined cases (74.1% vs. 59.1%). In the non-narrowly defined cases, more patients tended to visit the hospital before admission (within 90 days pre-admission, 61.7% vs. 70.3%).

## Discussion

This clinical study of 504 Japanese patients with AC elucidated clinical characteristics of AC. Patients with preexisting primary AI or central AI were not the majority of all patients. Indications for hospitalization and comorbidities on admission varied and tended to be associated with predisposing diseases. The proportion of admissions under oral GC treatment was only one-fifth of all admissions, and there were a small number of patients under treatment with inhaled GC or using interacting drugs.

Previous research on AC is limited and study populations mostly consisted of patients with chronic AI [3–5, 9, 16] or a critical illness [25]. In these studies, chronic AI indicated a diagnosis of AC as follows: an acute impairment of general health requiring hospital admission and necessity for administration of GC (Additional file 3: Table S12) [3–5]. Although our definition is similar to that definition, the clinical distinction between AI and AC is ambiguous. To describe the entire pictures of patients with AC, we initially selected data on patients with conditions consistent with AI, and then subsequently identified patients with AC based on their clinical management.

A previous cross-sectional study reported that AC occurred in 185 of 444 patients with chronic AI (primary adrenal insufficiency [PAI]: 119, secondary adrenal insufficiency [SAI]: 66) [5]. They showed that gastrointestinal infection was the most frequent precipitating factor (28.5%, 83/291), followed by other infectious diseases (21.6%, 63/291). Cessation of GC accounted for 7.6% (22/291) of cases. Risk factors for AC were primary AI, a concomitant disease, age at diagnosis, being female, and diabetes insipidus. In a cross-sectional study Ono et al. reported that AC occurred in 799 patients with chronic AI (PAI: 248, SAI; 551) [9]. They found that the most prevalent comorbidity was infection (15.0%) followed by respiratory diseases (11.8%). Two fifths of patients with PAI were less than 20 years and be significantly younger than patients with SAI. Rushworth et al. conducted a descriptive study of 824 adult patients admitted with AC [10] with the major comorbidity being infection (38.5%) and diabetes mellitus (21.6%). Predisposing chronic AI was reported in less than 20% of these patients.

In comparison with previous studies, our study showed a lower proportion of primary AI among participants with AC [5, 9]. One reason may be that the incidence of Addison’s disease in Japan is lower than in western countries [26], especially in the adult population [9]. It may also imply that the diagnosis of predisposing conditions is difficult and often delayed in Japan and elsewhere [17, 18]. Chronic AI is a risk factor for AC although there are many other risk factors. Critical illness, such as sepsis, tends to be complicated with AI [11]. Therefore, patients without a predisposing disease, that is those in the “Others” group, were affected by various illnesses including cancer, autoimmune disease, or renal failure and accounted for a major portion of AC patients.

Overall, three quarters of patients with AC in our database were aged 60 years or more. It may be notable that patients with “Others” were older and had more comorbidities associated with aging. Moreover, HPA axis function also alters during aging [27], which would influence the onset of AC. Older patients have a higher incidence of AC than younger patients and the condition tends to be more severe [9, 10]. Taken together, our results and those of previous studies highlight that clinicians should consider a patient’s age and comorbidities in addition to general status and predisposing conditions in the care of AC.

We found that cancer was both a major disease indicating admission as well as a comorbidity in our study, a result that could lead to several interpretations. Anticancer treatment requires the regular administration of GC drugs as antiemetic agents, which may suppress HPA axis function [28]. GC drugs are also effective as supportive care for cancer patients [29] and used frequently. Bilateral adrenal metastasis may induce AC. Lam et al. [30] reported that adrenal metastasis was detected in 3.1% of autopsied cancer patients, with half having bilateral metastasis. It is important to be aware of GC withdrawal and adrenal metastasis in the treatment of patient with cancer.

GC withdrawal could be a cause of AC, and prior studies reported that 4–8% of AC cases resulted from GC cessation [3–5]. In our study, a similar proportion of AC occurred within 30 days after GC cessation (36 patients, 7.1%). Adherence to treatment is recognized to be insufficient in some patients and is important from the viewpoint of risk management [31]. Therefore, we should improve patient education to decrease iatrogenic AC.

Recently, inhaled corticosteroid was indicated to increase the risk of AI [32, 33], but medication within standard doses was shown to have only a slight influence [33]. In our study, onset of AC under medication with inhaled corticosteroid was observed in only 15 cases (3.0%). Concomitant medication with interacting drugs was also limited, with only 13 cases (2.6%) identified.

This study has several limitations. First, administrative data have a weakness regarding rigidity of information on diseases. Therefore, we scrutinized diseases using DPC data, which represents the practical scenario of in-hospital treatment and identified diseases indicating admission and comorbidities on admission. Furthermore, we performed a sensitivity analysis to resolve some of the weaknesses of the use of administrative data. Second, we could not evaluate laboratory values such as serum cortisol levels. However, in practice it takes several days to get results of hormonal testing, and stimulating testing is not recommended in acute care settings [34]. Third, regarding assessment of prior treatment before admission, about 40% of the patients with AC were referred to the acute care hospitals from other institutions and we may have underestimated the proportion of patients under preadmission medications. Finally, clinical practice guidelines for AI and AC were just published in 2016 [23, 35], however, our data were from between 2003 and 2014. During that period, awareness of AC might have been poor in general practice setting. In our study fulfilling the criteria for AC involved both diagnosis and treatment. If both were not fulfilled, the number of patients with AC would have been much higher.

## Conclusions

Our study provided a pragmatic overview of patients with AC in acute care hospitals regardless of medical departments. Primary AI and central AI accounted for only one third of patients with AC, implying that the predisposing condition for AC is complicated and its diagnosis is often indeterminable or delayed. In addition to predisposing conditions, we should consider to patient’s age and comorbidities in the treatment of AC. Prospective epidemiological studies are needed to establish a standard protocol for prompt diagnosis of patients with AC.

## Additional files

Additional file 1: Table S1. Disease lists corresponding with adrenal insufficiency and number of patients. **Table S2.** Demographic and clinical characteristics at admission of patients receiving excessive GC administration (hydrocortisone equivalent dose >1000 mg/day). **Table S3.** Disease lists corresponding with risk factors for primary AI. **Table S4.** Diseases corresponding with risk factors of central AI. **Table S5.** Codes for pituitary disease, adrenal tumor and adrenal insufficiency (AI)-related symptoms as an indication for hospital admission. **Table S6.** Diseases corresponding with comorbidities. **Table S7.** Drugs that interact with glucocorticoids. **Table S8.** Comparison of glucocorticoid preparations. **Table S9.** Clinical characteristics in “Others” category at admission according to prior admission within 1 year before AC. **Table S10.** Clinical characteristics in “Others” category at admission with or without hospitalization under GC medication. **Table S11.** Clinical characteristics in “Others” category at admission according to hospitalization within 30 days after GC cessation. (DOCX 46 kb)

Additional file 2: Figure S1. Histograms showing interval between admission and disease registry date of AI. **Figure S2.** Histograms showing interval between admission and start of therapeutic GC administration. **Figure S3.** Distribution of hormone testing during hospitalization. (PPTX 220 kb)

Additional file 3: Table S12. Review of previous studies. (XLS 41 kb)

## Abbreviations

AC: Adrenal crisis
AI: Adrenal insufficiency
DPC: Diagnosis procedure combination
EphMRA: European pharmaceutical marketing research association
HPA: Hypothalamic-pituitary-adrenal
ICD-10: International statistical classification of diseases and related health problems 10th revision
IQR: Interquartile ranges
PAI: Primary adrenal insufficiency
SAI: Secondary adrenal insufficiency
