# Supplementary material for: Clinical characteristics of adrenal crisis in adult population with and without predisposing chronic adrenal insufficiency: a retrospective cohort study
Source: BMC Endocr Disord. 2017 Sep 11;17:58. doi: 10.1186/s12902-017-0208-0 (PMC5594557; doi:10.1186/s12902-017-0208-0)
Supplement: Supplementary file 1 — Disease lists corresponding with adrenal insufficiency and number of patients. Table S2. Demographic and clinical characteristics at admission of patients receiving excessive GC administration (hydrocortisone equivalent dose >1000 mg/day). Table S3. Disease lists corresponding with risk factors for primary AI. Table S4. Diseases corresponding with risk factors of central AI. Table S5. Codes for pituitary disease, adrenal tumor and adrenal insufficiency (AI)-related symptoms as an indication for hospital admission. Table S6. Diseases corresponding with comorbidities. Table S7. Drugs that interact with glucocorticoids. Table S8. Comparison of glucocorticoid preparations. Table S9. Clinical characteristics in “Others” category at admission according to prior admission within 1 year before AC. Table S10. Clinical characteristics in “Others” category at admission with or without hospitalization under GC medication. Table S11. Clinical characteristics in “Others” category at admission according to hospitalization within 30 days after GC cessation. (DOCX 46 kb) [file 12902_2017_208_MOESM1_ESM.docx]

| **Table S1.** Disease lists corresponding with adrenal insufficiency and number of patients. | |  |
| --- | --- | --- |
|  |  |  |
| Disease name | ICD-10 | Number of patients |
| Adrenocortical insufficiency | E274 | 264 |
| Adrenal crisis | E272 | 194 |
| Secondary adrenocortical insufficiency | E274 | 28 |
| Post-procedural adrenocortical hypofunction | E896 | 10 |
| Steroid withdrawal syndrome | E273 | 5 |
| Iatrogenic adrenocortical insufficiency | E274 / E896 | 3 |

| **Table S2.**  Demographic and clinical characteristics at admission of patients receiving excessive GC administration  (hydrocortisone equivalent dose > 1000 mg/day) | | | | | | | |
| --- | --- | --- | --- | --- | --- | --- | --- |
|  |  |  | HC > 1000mg/day | |  | ALL | |
|  |  |  | (n=41) | |  | (n=504) | |
| Age(years) | Median [IQR] |  | 64 | (54 - 75) |  | 71 | (59 - 80) |
|  |  |  |  |  |  |  |  |
| Male |  |  | 21 | (51.2) |  | 255 | (50.6) |
|  |  |  |  |  |  |  |  |
| Predisposing disease |  |  |  |  |  |  |  |
|  | Primary AI |  | 3 | (7.3) |  | 23 | (4.6) |
|  | Central AI |  | 11 | (26.8) |  | 136 | (27.0) |
|  | Others |  | 27 | (65.9) |  | 345 | (68.5) |
|  |  |  |  |  |  |  |  |
| Inidication for hospital admission* |  |  |  |  |  |  |  |
|  | Adrenal insufficiency† |  | 5 | (12.2) |  | 82 | (16.3) |
|  | Pituitary disease |  | 10 | (24.4) |  | 74 | (14.7) |
|  | Cancer |  | 2 | (4.9) |  | 74 | (14.7) |
|  | Infection |  | 0 | (0) |  | 59 | (11.1) |
|  | Cardiovascular disease |  | 3 | (7.3) |  | 21 | (4.2) |
|  | Sepsis |  | 2 | (4.9) |  | 17 | (3.4) |
|  | Adrenal tumor |  | 0 | (0) |  | 10 | (2.0) |
|  | AI-related clinical symptom‡ |  | 1 | (2.4) |  | 58 | (11.5) |
|  | Others |  | 14 | (34.6) |  | 86 | (17.1) |
|  |  |  |  |  |  |  |  |
| Therapeutic GC regimen |  |  |  |  |  |  |  |
|  | HC |  | 3 | (7.3) |  | 328 | (65.3) |
|  | mPSL |  | 37 | (90.2) |  | 74 | (14.7) |
|  | DEX |  | 0 | (0) |  | 44 | (8.7) |
|  | Others |  | 1 | (2.4) |  | 58 | (11.3) |
|  |  |  |  |  |  |  |  |
| Hormone testing |  |  |  |  |  |  |  |
|  | ACTH |  | 17 | (41.5) |  | 234 | (46.4) |
|  | Cortisol |  | 23 | (56.1) |  | 258 | (51.2) |
|  | Endocrine stimulation test§ |  | 3 | (7.3) |  | 40 | (7.9) |
|  | Adrenal cortex stimulation test |  | 0 | (0) |  | 21 | (4.2) |
|  |  |  |  |  |  |  |  |
| Visited before admission |  |  |  |  |  |  |  |
|  | Yes, within 90 days before |  | 26 | (63.4) |  | 337 | (66.9) |
|  | Yes, within 14 days before |  | 20 | (48.8) |  | 241 | (47.8) |
|  |  |  |  |  |  |  |  |
| Under GC-related medication |  |  |  |  |  |  |  |
|  | Oral GC |  | 9 | (22.0) |  | 104 | (20.6) |
|  | HC equivalent daily dosage (mg) |  | 30 | (21 - 157) |  | 27 | (20 - 100) |
|  | Median [IQR] |  |  |  |  |  |  |
|  |  |  |  |  |  |  |  |
|  | Intranasal GC |  | 0 | (0.0) |  | 0 | (0.0) |
|  | Inhaled GC |  | 2 | (4.9) |  | 15 | (3.0) |
|  | Drug interacting with GC |  | 1 | (2.4) |  | 13 | (2.6) |
| Abbreviation:  AI, adrenal insufficiency; GC, glucocorticoid; HC, hydrocortisone; mPSL, methylprednisolone; DEX, dexamethasone; ACTH, adrenocorticotropic hormone; IQR, Interquartile  * Identified as disease or symptom requiring decision to admit registered in the DPC system. † Considering with following admission and therapeutic GC administration, this group is consistent with hospitalization due to adrenal crisis. ‡ Consisted of unspecified coma, hyponatremia, unspecified hypotension, volume depletion, shock, anorexia, nausea, vomiting, unspecified fever and hypoglycemia. § Endocrine stimulation test consists of hormone dynamic testing of the following: anterior pituitary (growth hormone, gonadotropin, thyroid stimulating hormone, prolactin, adrenocorticotropic hormone), posterior pituitary (antidiuretic hormone), thyroid, parathyroid, and gonad (testosterone, estradiol). Endocrine tests of adrenocorticotropic hormone included insulin tolerance test, metyrapone test, dexamethasone suppression test, and corticotropin-releasing hormone stimulation test. In this study, we counted results of the adrenal cortex stimulation test apart from those for endocrine stimulation tests. The adrenal stimulation tests evaluates adrenal cortex function, which is related to glucocorticoid or mineralocorticoid, for example, the adrenocorticotropic hormone stimulation test. | | | | | | | |
|  |  |  |  |  |  |  |  |
|  |  |  |  |  |  |  |  |
|  |  |  |  |  |  |  |  |
|  |  |  |  |  |  |  |  |
|  |  |  |  |  |  |  |  |
|  |  |  |  |  |  |  |  |
|  |  |  |  |  |  |  |  |
|  |  |  |  |  |  |  |  |
|  |  |  |  |  |  |  |  |
|  |  |  |  |  |  |  |  |
|  |  |  |  |  |  |  |  |
|  |  |  |  |  |  |  |  |
|  |  |  |  |  |  |  |  |
|  |  |  |  |  |  |  |  |

| **Table S3.** Disease lists corresponding with risk factors for primary AI. | |  |  |  |  |
| --- | --- | --- | --- | --- | --- |
|  |  |  |  |  |  |
| Riskfactor of primary AI disease | ICD-10 (ver.2008) | Surgical intervention needed |  | Surgical intervention of adrenal gland | Surgical procedure codes (Japanese original) |
| Tuberculous adrenalitis | A187 |  |  | Adrenalectomy | K754/K755/K756 |
| Waterhouse–Friderichsen syndrome | A391 |  |  | Laparoscopic adrenalectomy | K754-2/K7547-3/K756-2 |
| Congenital adrenal hyperplasia | E250 |  |  |  |  |
| 21-hydroxylase deficiency |  |  |  |  |  |
| 11β-hydroxylase deficiency |  |  |  |  |  |
| 3β-hydroxysteroid dehydrogenase type 2 deficiency |  |  |  |  |  |
| 17α-hydroxylase deficiency |  |  |  |  |  |
| P450 oxidoreductase deficiency |  |  |  |  |  |
| Congenital lipoid adrenal hyperplasia |  |  |  |  |  |
| Autoimmune adrenalitis | E271 |  |  |  |  |
| Addison’s disease |  |  |  |  |  |
| Adrenal haemorrhage | E274 |  |  |  |  |
| Adrenal infarction | E274 |  |  |  |  |
| Adrenal hypoplasia congenital(X-linked) | E274 |  |  |  |  |
| Adrenal hypoplasia congenital(SF-1 linked) | E274 |  |  |  |  |
| IMAGe syndrome | E274 |  |  |  |  |
| Triple A syndrome (Allgrove’s syndrome) | E274 |  |  |  |  |
| ACTH insensitivity syndrome | E274 |  |  |  |  |
| Adrenalitis | E278 |  |  |  |  |
| Autoimmune polyendocrinopathy syndrome(APS) | E310 |  |  |  |  |
| APS type1 |  |  |  |  |  |
| APS type2 |  |  |  |  |  |
| APS type3 |  |  |  |  |  |
| Adrenal hypothyrodism | E310 |  |  |  |  |
| Adrenoleukodystrophy | E713 |  |  |  |  |
| Adrenomyeloneuropathy | E713 |  |  |  |  |
| Postprocedural adrenocortical(-medullary) hypofunction | E896 |  |  |  |  |
|  |  |  |  |  |  |
|  |  |  |  |  |  |
| Malignant neoplasm of adrenal gland | C74 | Yes |  |  |  |
| Secondary malignant neoplasm of adrenal gland | C797 | Yes |  |  |  |
| Benign neoplasm of adrenal gland | D350 | Yes |  |  |  |
| Neoplasm of adrenal gland | D441 | Yes |  |  |  |
| Primary hyperaldosteronism | E260 | Yes |  |  |  |
| Injury of adrenal gland | S378 | Yes |  |  |  |

| **Table S4.** Diseases corresponding with risk factors of central AI. | |  |  |  |  |
| --- | --- | --- | --- | --- | --- |
|  |  |  |  |  |  |
| Riskfactor of secondary AI disease | ICD-10 (ver.2008) | Surgical intervention needed |  | Surgical intervention of hypothalamus or pituitary | Surgical procedure codes (Japanese original) |
| **Pituitary tumors** |  |  |  | Endonasal trans-sphenoidal pituitary surgery | K171 |
| Germinoma | C719 | Yes |  | Endoscopic endonasal trans-sphenoidal pituitary surgery | K171-2 |
| Meningioma | D320 | Yes |  | Intracranial surgery | K167/K169 |
| Adenoma |  | D352 |  |  |  |
| Other malignant neoplasms | C751 | Yes |  |  |  |
| Other pathological neoplasms | D443 | Yes |  |  |  |
| Craniopharyngioma | D353 | Yes |  |  |  |
| Malignant neoplasms of craniopharyngeal duct | C752 | Yes |  |  |  |
| Other pathological neoplasms of craniopharyngeal duct | D444 | Yes |  |  |  |
| **Hypothalamic tumors** |  |  |  |  |  |
| Astrocytoma | C710 | Yes |  |  |  |
| Other pathologic neoplasms | D430 | Yes |  |  |  |
| **Parasellar tumors** |  |  |  |  |  |
| Parasellar epidermoid cyst | D339 | Yes |  |  |  |
|  |  |  |  |  |  |
| Acromegaly and pituitary gigantism | E220 | Yes |  |  |  |
| Hyperprolactinaemia | E221 | Yes |  |  |  |
| Rathke’s cleft cyst | E236 | Yes |  |  |  |
| Cushing’s disease | E240 | Yes |  |  |  |
|  |  |  |  |  |  |
| Panhypopituitarism after surgery and/or radiation | E230 |  |  |  |  |
| Isolated ACTH deficiency | E230 |  |  |  |  |
| Idiopathic hypopituitarism | E230 |  |  |  |  |
| Sheehan’s syndrome | E230 |  |  |  |  |
| Hypothalamic dysfunction | E233 |  |  |  |  |
| Empty sella syndrome | E236 |  |  |  |  |
| Lymphocytic hypophysitis | E236 |  |  |  |  |
| Pituitary apoplexy | E236 |  |  |  |  |
| Pituitary dysfunction | E237 |  |  |  |  |
| Postprocedural hypopituitarism | E893 |  |  |  |  |
| Postirradiation hypopituitarism | E893 |  |  |  |  |
| Prader-Willi syndrome | Q871 |  |  |  |  |

| **Table S5.** Codes for pituitary disease, adrenal tumor and adrenal insufficiency (AI)-related symptoms as an indication for hospital admission. | |
| --- | --- |
|  |  |
| Disease indicating admission | ICD-10 (ver.2008) |
| Pituitary disease | D352, D353, D443, D444, E230, E236, D353 |
| Adrenal disease | D350, D441 |
| AI-related symptom | R402, E871, I959, E86, R57, R630, R11, R509, E15, E160-E162 |

| **Table S6.** Diseases corresponding with comorbidities. | |
| --- | --- |
|  |  |
| Comorbidity | ICD-10 (ver.2008) |
| Cardiovascular disease | I05-I15, I20-I26, I50, I60-I74, I80, Q20-Q28, G45-G46 |
| Malignant neoplasm | C00-C97 |
| Diabetes | E10-E14 |
| COPD or asthma | J41-J46 |
| Infectious disease | A00-A39, A42-A99, B00-B17, B19-B99, G00-G07, I00-I02, I30.1, I32.0, I33, I40.0, J00-J12, J13-J22, J36, J39.0, J85-J86, K35-K37, K57.0, K57.2, K57.8, K61, K63.0, K65, K67, K75.0, K80.0, K80.3-K80.4, K81.9, K83.0, L00-L03, L05-L08, M00-M01, M86, N10, N34.0, N39.0, N41 |
| Renal failure | N17-N18 |
| Liver disease | K70-K74.2, K74.4-K74.6. B18 |
| Osteoporosis | M80-M82 |
| Sepsis | A40-A41 |
| Dementia | F00-F04 |
| Peptic ulcer | K25-K28 |
| Autoimmune disease | D51.0, D59.1, D69.3, D86, E05.0, E06.3, G35, G70.0, H20.0-H20.1, J84.1, K50-K51, K74.3, K75.4, K90.0, L10, L12, L13.0, L20, L40, L80, M05-M07.3, M08, M30-M35.3, M35.6, M45 |
| Hypothyrodism | E00-E03 |
|  |  |
|  |  |
| Abbreviation: COPD; chronic obstructive pulmonary disease | |
|  |  |
| Autoimmune diseases are as follows;  Autoimmune hemolytic anemia, idiopathic thrombocytopenic purpura, Graves’disease, autoimmune thyroiditis, multiple sclerosis, myasthenia gravis, pernicious anemia, celiac disease, Crohn disease, ulcerative colitis,  autoimmune hepatitis, atopic dermatitis, pemphigus, pemphigoid, herpetiform dermatitis, psoriasis, leukodermia, rheumatoid arthritis, juvenile rheumatoid arthritis, spondylarthritis ankylopoietica, polymyositis, dermatomyositis,  scleroderma, systemic lupus erythematosus, mixed connective tissue disease, Sjoegren syndrome, sarcoidosis, vasculitis, polymyalgia rheumatica, psoriatic arthritis, Behcet’s disease, idiopathic pulmonary fibrosis and iridocyclitis | |

| **Table S7.** Drugs that interact with glucocorticoids. |  |
| --- | --- |
|  |  |
| Interacting medications with systemic glucocorticoid | EphMRA-ATC |
| **Anticonvulsants** |  |
| Barbiturates | A03C, A03D, N05B3, N05B4 |
| Primidone, Carbamazepine, Phenytoin | N03A0 |
|  |  |
| **Hormonal medications** |  |
| Estrogens | G03A1, G03A2, D07B1, G03C, L02A1 |
| Estramustine phosphate sodium hydrate | L01X9 |
| Estrogen with progesterone combinations | G03A3, G03A4, G03F |
| Androgen with female hormone combinations | G03E |
| Progesterones | G03D, G03S5, L02A2 |
|  |  |
| **Antibiotics** |  |
| Erythromycin | J01F0 |
| Rifamycins | J01K, J01L, J03A, J01M |
| **Antifungals** |  |
| Ketoconazole | D01A1 |
| Itraconazole | J02A |
| **Antivirals** |  |
| Ritonavir | J05C2 |
|  |  |
| **Others** |  |
| Ephedrine | R05D1 |
| Cyclosporine | L04X0 |
| Chlorpromazine | N05A9 |
| Imipramine | N06A9 |
| Topiramate | N03A0 |
| Mitotane | L01X9 |

| **Table S8.** Comparison of glucocorticoid preparations. | |
| --- | --- |
|  |  |
| Glucocorticoid category | Equivalent doses (mg) |
| Hydrocortisone (cortisol) | 20 |
| Cortisone acetate | 25 |
| Prednisone | 5 |
| Prednisolone | 5 |
| Methylprednisolone | 4 |
| Triamcinolone | 4 |
| Fludrocortisone | Not used for an antiinflammatory effect |
| Dexamethasone | 1 |

| **Table S9.** Clinical characteristics in "Others" category at admission according to prior admission within 1 year before AC. | | | | | | | |
| --- | --- | --- | --- | --- | --- | --- | --- |
|  |  |  | Prior admission within 1 year before AC | | | | |
|  |  |  | Yes |  |  | No |  |
|  |  |  | (N=152) |  |  | (N=193) |  |
| Indication for hospital admission* | | |  |  |  |  |  |
|  | Adrenal insufficiency† | | 23 | (15.1) |  | 27 | (14.0) |
|  | Pituitary disease | | 1 | (0.7) |  | 1 | (0.5) |
|  | Cancer |  | 39 | (25.7) |  | 30 | (15.5) |
|  | Infection |  | 17 | (11.2) |  | 27 | (14.0) |
|  | Cardiovascular disease | | 2 | (1.3) |  | 17 | (8.8) |
|  | Sepsis |  | 4 | (2.6) |  | 9 | (4.7) |
|  | Adrenal disease | | 7 | (4.6) |  | 3 | (1.6) |
|  | AI related clinical symptom‡ | | 19 | (12.5) |  | 25 | (13.0) |
|  |  |  |  |  |  |  |  |
| Comorbidity§ | |  |  |  |  |  |  |
|  | Cardiovascular disease | | 45 | (29.6) |  | 64 | (33.2) |
|  | Infection |  | 27 | (17.8) |  | 38 | (19.7) |
|  | Diabetes |  | 27 | (17.8) |  | 30 | (15.5) |
|  | Cancer |  | 41 | (27.0) |  | 25 | (13.0) |
|  | Hypothyroidism | | 6 | (3.9) |  | 18 | (9.3) |
|  | Autoimmune disease | | 18 | (11.8) |  | 14 | (7.3) |
|  | Peptic ulcer |  | 10 | (6.6) |  | 11 | (5.7) |
|  | COPD or asthma | | 11 | (7.2) |  | 10 | (5.2) |
|  | Renal failure |  | 8 | (5.3) |  | 13 | (6.7) |
|  |  |  |  |  |  |  |  |
| Hormone testing | |  |  |  |  |  |  |
|  | ACTH |  | 48 | (31.6) |  | 79 | (40.9) |
|  | Cortisol |  | 58 | (38.2) |  | 86 | (44.6) |
|  | Endocrine stimulation test\|\| | | 3 | (2.0) |  | 6 | (3.1) |
|  | Adrenal cortex stimulation test\|\| | | 2 | (1.3) |  | 7 | (3.6) |
|  |  |  |  |  |  |  |  |
| Hormone testing before AC | | |  |  |  |  |  |
|  | ACTH |  | 43 | (28.3) |  | 11 | (5.7) |
|  | Cortisol |  | 47 | (30.9) |  | 12 | (6.2) |
|  | Endocrine stimulation test\|\|\| | | 2 | (1.3) |  | 0 |  |
|  | Adrenal cortex stimulation test\|\| | | 4 | (2.6) |  | 2 | (1.0) |
|  |  |  |  |  |  |  |  |
| Hospital referral | |  | 59 | (38.8) |  | 83 | (43.0) |
|  |  |  |  |  |  |  |  |
| Under GC-related medication | | |  |  |  |  |  |
|  | Oral GC |  | 45 | (29.6) |  | 23 | (11.9) |
|  | HC equivalent daily dosage (mg) | | 20 | (20 - 40) |  | 26.7 | (20 - 56.7) |
|  | Median [IQR] | |  |  |  |  |  |
|  |  |  |  |  |  |  |  |
|  | Inhaled GC |  | 8 | (5.3) |  | 6 | (3.1) |
|  | Drug interacting with GC | | 3 | (2.0) |  | 5 | (2.6) |
|  |  |  |  |  |  |  |  |
| Timelag between admission and GC cessation | | |  |  |  |  |  |
|  | Within 14 days after GC cessation | | 18 | (11.8) |  | 3 | (1.6) |
|  | Within 30 days after GC cessation | | 28 | (18.4) |  | 3 | (1.6) |
| Abbreviations: AC, adrenal crisis; GC, glucocorticoid; AI, adrenal insufficiency; COPD, chronic obstructive pulmonary disease; ACTH, adrenocorticotropic hormone * Identified as disease or symptom requiring admission decision, registered according to the Japanese diagnostic procedure combination (DPC) system. † Considering with following admission and therapeutic GC administration, this group is consistent with hospitalization due to adrenal crisis. ‡ Consisted of unspecified coma, hyponatremia, unspecified hypotension, volume depletion, shock, anorexia, nausea, vomiting, unspecified fever and hypoglycemia. § Identification of comorbidity is based on comorbidity lists at admission, registered according to the Japanese diagnostic procedure combination (DPC) system.  \|\| Endocrine stimulation test consists of hormone dynamic testing including the following: anterior pituitary (growth hormone, gonadotropin, thyroid stimulating hormone, prolactin, adrenocorticotropic hormone), posterior pituitary (antidiuretic hormone), thyroid, parathyroid, and gonad (testosterone, estradiol). Endocrine tests of adrenocorticotropic hormone included insulin tolerance test, metyrapone test, dexamethasone suppression test, and corticotropin-releasing hormone stimulation test. In this study, we counted results of adrenal cortex stimulation test apart from these endocrine stimulation tests. The adrenal stimulation tests evaluates adrenal cortex function, which is related to glucocorticoid or mineralocorticoid, for example, the adrenocorticotropic hormone stimulation test. | | | | | | | |
|  |  |  |  |  |  |  |  |
|  |  |  |  |  |  |  |  |
|  |  |  |  |  |  |  |  |
|  |  |  |  |  |  |  |  |
|  |  |  |  |  |  |  |  |
|  |  |  |  |  |  |  |  |
|  |  |  |  |  |  |  |  |
|  |  |  |  |  |  |  |  |
|  |  |  |  |  |  |  |  |
|  |  |  |  |  |  |  |  |
|  |  |  |  |  |  |  |  |
|  |  |  |  |  |  |  |  |
|  |  |  |  |  |  |  |  |
|  |  |  |  |  |  |  |  |
|  |  |  |  |  |  |  |  |
|  |  |  |  |  |  |  |  |

| **Table S10.** Clinical characteristics in "Others" category at admission with or without hospitalization under GC medication. | | | | | | | |
| --- | --- | --- | --- | --- | --- | --- | --- |
|  |  |  | Hospitalized for AC under GC medication | | | | |
|  |  |  | Yes |  |  | No |  |
|  |  |  | (N=80) |  |  | (N=265) |  |
| Indication for hospital admission* | | |  |  |  |  |  |
|  | Adrenal insufficiency† | | 8 | (10.0) |  | 42 | (15.8) |
|  | Pituitary disease | | 0 |  |  | 2 | (0.8) |
|  | Cancer |  | 18 | (22.5) |  | 51 | (19.2) |
|  | Infection |  | 15 | (18.8) |  | 29 | (10.9) |
|  | Cardiovascular disease | | 1 | (1.3) |  | 18 | (6.8) |
|  | Sepsis |  | 4 | (5.0) |  | 9 | (3.4) |
|  | Adrenal disease | | 0 |  |  | 10 | (3.8) |
|  | AI-related clinical symptom‡ | | 13 | (16.3) |  | 31 | (11.7) |
|  |  |  |  |  |  |  |  |
| Comorbidity§ | |  |  |  |  |  |  |
|  | Cardiovascular disease | | 24 | (30.0) |  | 85 | (32.1) |
|  | Infection |  | 12 | (15.0) |  | 53 | (20.0) |
|  | Diabetes |  | 10 | (12.5) |  | 47 | (17.7) |
|  | Cancer |  | 22 | (27.5) |  | 44 | (16.6) |
|  | Hypothyroidism | | 3 | (3.8) |  | 21 | (7.9) |
|  | Autoimmune disease | | 13 | (16.3) |  | 19 | (7.2) |
|  | Peptic ulcer |  | 7 | (8.8) |  | 14 | (5.3) |
|  | COPD or asthma | | 8 | (10.0) |  | 13 | (4.9) |
|  | Renal failure |  | 3 | (3.8) |  | 18 | (6.8) |
|  |  |  |  |  |  |  |  |
| Hormone testing | |  |  |  |  |  |  |
|  | ACTH |  | 22 | (27.5) |  | 105 | (39.6) |
|  | Cortisol |  | 29 | (36.3) |  | 115 | (43.4) |
|  | Endocrine stimulation test\|\| | | 0 |  |  | 9 | (3.4) |
|  | Adrenal cortex stimulation test\|\| | | 2 | (2.5) |  | 7 | (2.6) |
|  |  |  |  |  |  |  |  |
| Hormone testing before AC | | |  |  |  |  |  |
|  | ACTH |  | 22 | (27.5) |  | 32 | (12.1) |
|  | Cortisol |  | 25 | (31.3) |  | 34 | (12.8) |
|  | Endocrine stimulation test\|\| | | 1 | (1.3) |  | 1 | (0.4) |
|  | Adrenal cortex stimulation test\|\| | | 3 | (3.8) |  | 3 | (1.1) |
|  |  |  |  |  |  |  |  |
| Hospital referral | |  | 25 | (31.3) |  | 117 | (44.2) |
|  |  |  |  |  |  |  |  |
| Admission within 1 year before AC | | | 52 | (65.0) |  | 100 | (37.7) |
| Abbreviations: AC, adrenal crisis; GC, glucocorticoid; AI, adrenal insufficiency; COPD, chronic obstructive pulmonary disease; ACTH, adrenocorticotropic hormone * Identified as disease or symptom requiring admission registered according to the Japanese diagnostic procedure combination (DPC) system. † Considering with following admission and therapeutic GC administration, this group is consistent with hospitalization due to adrenal crisis. ‡ Consisted of unspecified coma, hyponatremia, unspecified hypotension, volume depletion, shock, anorexia, nausea, vomiting, unspecified fever and hypoglycemia. § Identification of comorbidity is based on comorbidity lists at admission, registered according to the Japanese diagnostic procedure combination (DPC) system.  \|\| Endocrine stimulation test consists of hormone dynamic testing including the following: anterior pituitary (growth hormone, gonadotropin, thyroid stimulating hormone, prolactin, adrenocorticotropic hormone), posterior pituitary (antidiuretic hormone), thyroid, parathyroid, and gonad (testosterone, estradiol). Endocrine tests of adrenocorticotropic hormone included insulin tolerance test, metyrapone test, dexamethasone suppression test, and corticotropin-releasing hormone stimulation test. In this study, we counted results of adrenal cortex stimulation test apart from these endocrine stimulation tests. The adrenal stimulation tests evaluates adrenal cortex function, which is related to glucocorticoid or mineralocorticoid, for example, the adrenocorticotropic hormone stimulation test. | | | | | | | |
|  |  |  |  |  |  |  |  |
|  |  |  |  |  |  |  |  |
|  |  |  |  |  |  |  |  |
|  |  |  |  |  |  |  |  |
|  |  |  |  |  |  |  |  |
|  |  |  |  |  |  |  |  |
|  |  |  |  |  |  |  |  |
|  |  |  |  |  |  |  |  |
|  |  |  |  |  |  |  |  |
|  |  |  |  |  |  |  |  |
|  |  |  |  |  |  |  |  |
|  |  |  |  |  |  |  |  |
|  |  |  |  |  |  |  |  |
|  |  |  |  |  |  |  |  |
|  |  |  |  |  |  |  |  |
|  |  |  |  |  |  |  |  |

| **Table S11.**  Clinical characteristics in "Others" category at admission according to hospitalization within 30 days after GC cessation. | | | | | | | |
| --- | --- | --- | --- | --- | --- | --- | --- |
|  |  |  | Hospitalized for AC within 30 days after GC cessation | | | | |
|  |  |  | Yes |  |  | No |  |
|  |  |  | (N=31) |  |  | (N=314) |  |
| Indication for hospital admission* | | |  |  |  |  |  |
|  | Adrenal insufficiency† | | 6 | (19.4) |  | 44 | (14.0) |
|  | Pituitary disease | | 0 |  |  | 2 | (0.6) |
|  | Cancer |  | 7 | (22.6) |  | 62 | (19.7) |
|  | Infection |  | 1 | (3.2) |  | 43 | (13.7) |
|  | Cardiovascular disease | | 1 | (3.2) |  | 18 | (5.7) |
|  | Sepsis |  | 0 |  |  | 13 | (4.1) |
|  | Adrenal disease | | 1 | (3.2) |  | 9 | (2.9) |
|  | AI related clinical symptom‡ | | 6 | (19.4) |  | 38 | (12.1) |
|  |  |  |  |  |  |  |  |
| Comorbidity§ | |  |  |  |  |  |  |
|  | Cardiovascular disease | | 9 | (29.0) |  | 100 | (31.8) |
|  | Infection |  | 6 | (19.4) |  | 59 | (18.8) |
|  | Diabetes |  | 5 | (16.1) |  | 52 | (16.6) |
|  | Cancer |  | 9 | (29.0) |  | 57 | (18.2) |
|  | Hypothyroidism | | 2 | (6.5) |  | 22 | (7.0) |
|  | Autoimmune disease | | 0 |  |  | 32 | (10.2) |
|  | Peptic ulcer |  | 3 | (9.7) |  | 18 | (5.7) |
|  | COPD or asthma | | 4 | (12.9) |  | 17 | (5.4) |
|  | Renal failure |  | 2 | (6.5) |  | 19 | (6.1) |
|  |  |  |  |  |  |  |  |
| Hormone testing | |  |  |  |  |  |  |
|  | ACTH |  | 9 | (29.0) |  | 118 | (37.6) |
|  | Cortisol |  | 12 | (38.7) |  | 132 | (42.0) |
|  | Endocrine stimulation test\|\| | | 0 |  |  | 9 | (2.9) |
|  | Adrenal cortex stimulation test\|\| | | 0 |  |  | 9 | (2.9) |
|  |  |  |  |  |  |  |  |
| Hormone testing before AC | | |  |  |  |  |  |
|  | ACTH |  | 11 | (35.5) |  | 43 | (13.7) |
|  | Cortisol |  | 13 | (41.9) |  | 46 | (14.6) |
|  | Endocrine stimulation test\|\| | | 0 |  |  | 2 | (0.6) |
|  | Adrenal cortex stimulation test\|\| | | 0 |  |  | 6 | (1.9) |
|  |  |  |  |  |  |  |  |
| Hospital referral | |  | 17 | (54.8) |  | 125 | (39.8) |
|  |  |  |  |  |  |  |  |
| Admission within 1 year before AC | | | 28 | (90.3) |  | 124 | (36.3) |
| Abbreviations: AC, adrenal crisis; GC, glucocorticoid; AI, adrenal insufficiency; COPD, chronic obstructive pulmonary disease; ACTH, adrenocorticotropic hormone * Identified as disease or symptom requiring admission registered according to the Japanese diagnostic procedure combination (DPC) system. † Considering with following admission and therapeutic GC administration, this group is consistent with hospitalization due to adrenal crisis. ‡ Consisted of unspecified coma, hyponatremia, unspecified hypotension, volume depletion, shock, anorexia, nausea, vomiting, unspecified fever and hypoglycemia. § Identification of comorbidity is based on comorbidity lists at admission, registered according to the Japanese diagnostic procedure combination (DPC) system.  \|\| Endocrine stimulation test consists of hormone dynamic testing including the following: anterior pituitary (growth hormone, gonadotropin, thyroid stimulating hormone, prolactin, adrenocorticotropic hormone), posterior pituitary (antidiuretic hormone), thyroid, parathyroid, and gonad (testosterone, estradiol). Endocrine tests of adrenocorticotropic hormone included insulin tolerance test, metyrapone test, dexamethasone suppression test, and corticotropin-releasing hormone stimulation test. In this study, we counted results of adrenal cortex stimulation test apart from these endocrine stimulation tests. The adrenal stimulation tests evaluates adrenal cortex function, which is related to glucocorticoid or mineralocorticoid, for example, the adrenocorticotropic hormone stimulation test. | | | | | | | |
|  |  |  |  |  |  |  |  |
|  |  |  |  |  |  |  |  |
|  |  |  |  |  |  |  |  |
|  |  |  |  |  |  |  |  |
|  |  |  |  |  |  |  |  |
|  |  |  |  |  |  |  |  |
|  |  |  |  |  |  |  |  |
|  |  |  |  |  |  |  |  |
|  |  |  |  |  |  |  |  |
|  |  |  |  |  |  |  |  |
|  |  |  |  |  |  |  |  |
|  |  |  |  |  |  |  |  |
|  |  |  |  |  |  |  |  |
|  |  |  |  |  |  |  |  |
|  |  |  |  |  |  |  |  |
|  |  |  |  |  |  |  |  |
