# Supplementary material for: Clinical characteristics of adrenal crisis in adult population with and without predisposing chronic adrenal insufficiency: a retrospective cohort study
Source: BMC Endocr Disord. 2017 Sep 11;17:58. doi: 10.1186/s12902-017-0208-0 (PMC5594557; doi:10.1186/s12902-017-0208-0)
Supplement: Supplementary file 2 — Histograms showing interval between admission and disease registry date of AI. Figure S2. Histograms showing interval between admission and start of therapeutic GC administration. Figure S3. Distribution of hormone testing during hospitalization. (PPTX 220 kb) [file 12902_2017_208_MOESM2_ESM.pptx]

## Slide 1
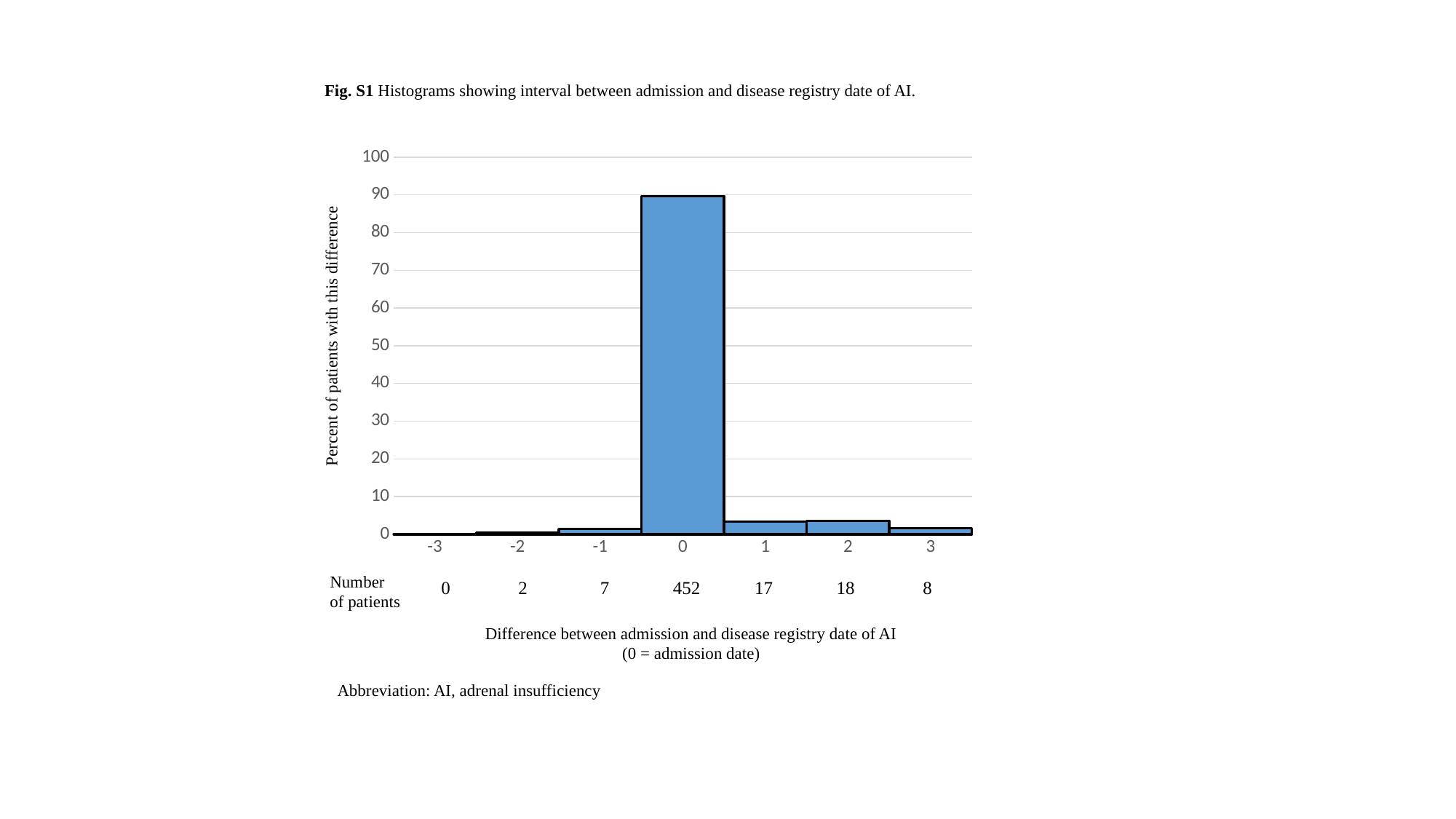

Fig. S1 Histograms showing interval between admission and disease registry date of AI.
### Chart
| Category | |
|---|---|
| -3 | 0.0 |
| -2 | 0.4 |
| -1 | 1.4 |
| 0 | 89.7 |
| 1 | 3.4 |
| 2 | 3.6 |
| 3 | 1.6 |Percent of patients with this difference
Number
of patients
0 2 7 452 17 18 8
Difference between admission and disease registry date of AI
(0 = admission date)
Abbreviation: AI, adrenal insufficiency

## Slide 2
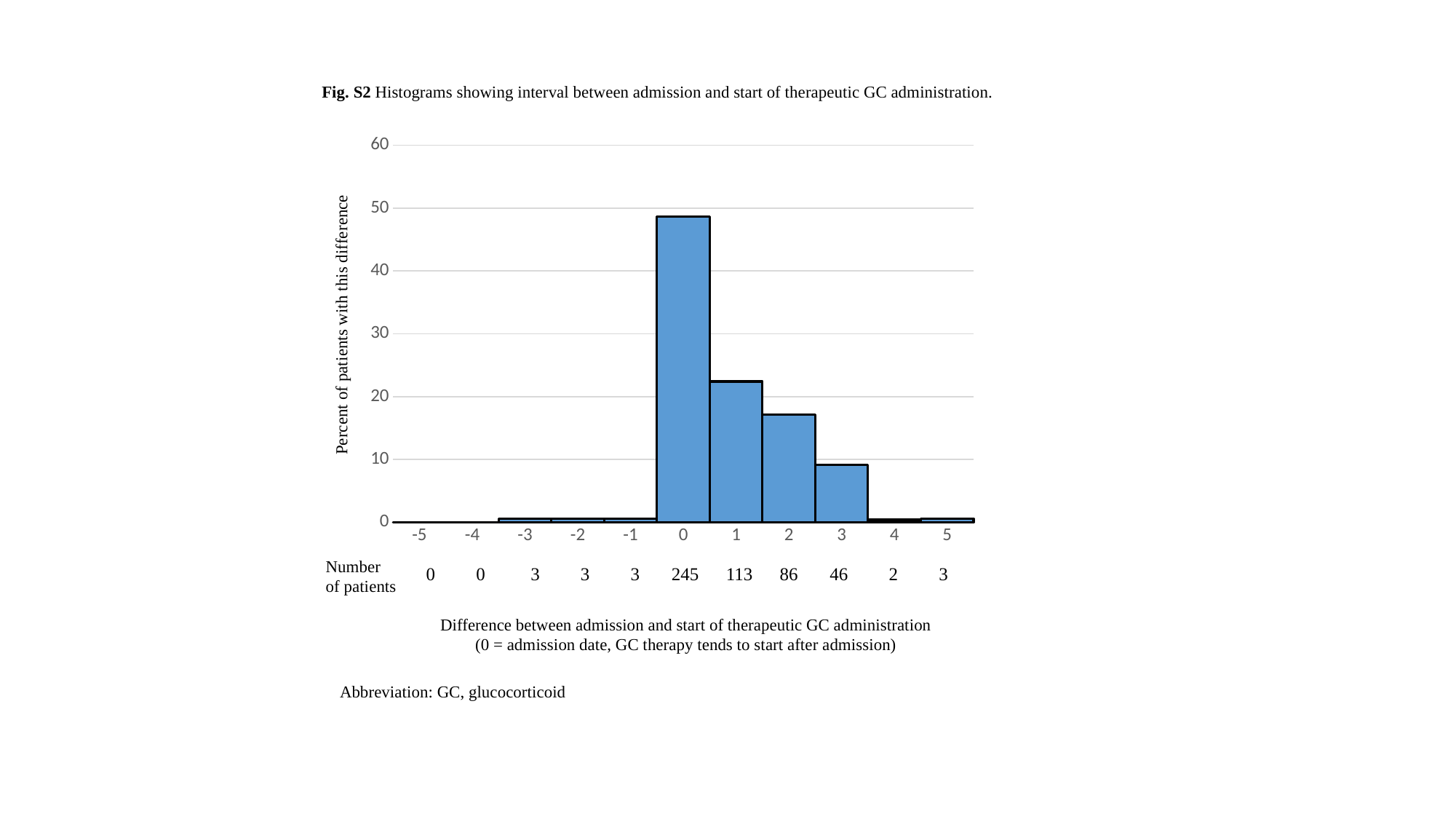

Fig. S2 Histograms showing interval between admission and start of therapeutic GC administration.
### Chart
| Category | |
|---|---|
| -5 | 0.0 |
| -4 | 0.0 |
| -3 | 0.6 |
| -2 | 0.6 |
| -1 | 0.6 |
| 0 | 48.6 |
| 1 | 22.4 |
| 2 | 17.1 |
| 3 | 9.1 |
| 4 | 0.4 |
| 5 | 0.6 |Percent of patients with this difference
Number
of patients
0 0 3 3 3 245 113 86 46 2 3
Difference between admission and start of therapeutic GC administration
(0 = admission date, GC therapy tends to start after admission)
Abbreviation: GC, glucocorticoid

## Slide 3
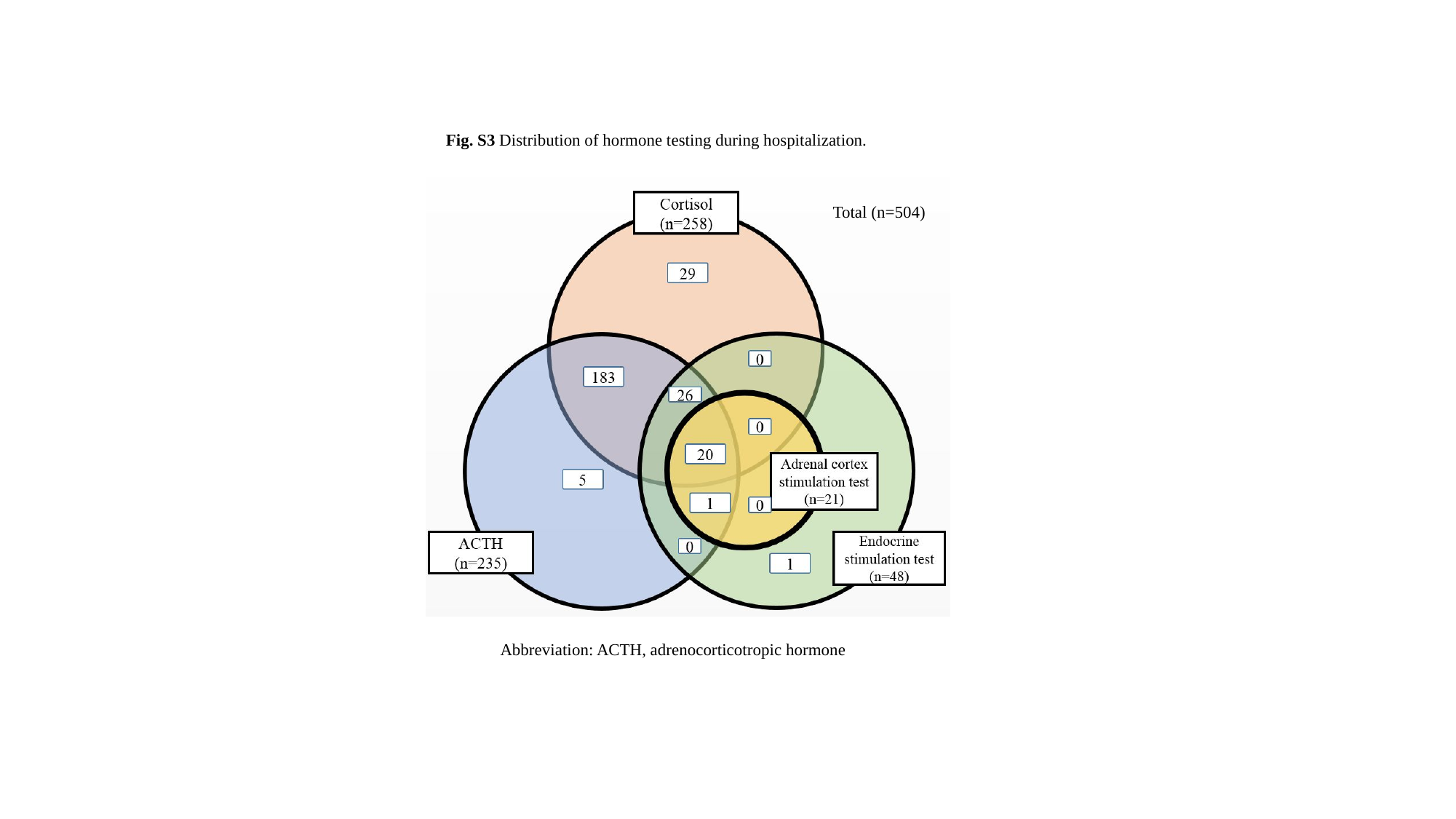

Fig. S3 Distribution of hormone testing during hospitalization.
Total (n=504)
Abbreviation: ACTH, adrenocorticotropic hormone
